# Recruiting patients for falls prevention in the emergency department – worth the challenge

**DOI:** 10.1186/s12877-023-04607-5

**Published:** 2023-12-21

**Authors:** Tim Stuckenschneider, Laura Schmidt, Elisa-Marie Speckmann, Jessica Koschate, Tania Zieschang

**Affiliations:** Department for Health Services Research, Geriatric Medicine, School of Medicine and Health Services, Carl von Ossietzky University, Ammerländer Heerstraße 114-118, Lower Saxony, 26129 Oldenburg, Germany

**Keywords:** Falls, Recruitment, Emergency department, Observational study, Older people

## Abstract

**Background:**

Severe falls escalate the risk of future falls and functional decline as indicated by recent global guidelines. To establish effective falls prevention, individuals at highest risk must be thoroughly studied and, therefore, successfully recruited.

**Objective:**

Recruiting from an emergency department (ED) may mitigate common selection biases, such as overrepresentation of individuals with a higher social status and healthier lifestyle. However, this approach presents unique challenges due to ED-specific conditions. Hence, we present the successes and challenges of an ED-based recruitment for an observational study.

**Methods:**

The SeFallED study targets older adults aged ≥60 years, who present to either of two hospitals in Oldenburg after a fall without subsequent admission. A study nurse addressed individuals in the EDs. Subsequently, potential participants were contacted by phone to arrange a home visit for obtaining written consent. Data of participants were compared with total admissions during the recruitment period to determine recruitment rate and compare patients’ characteristics.

**Results:**

Over 1.500 individuals met the inclusion criteria. Of these, 288 participants were successfully recruited. Most patients presented to the ED outside of the study team’s working hours, and some opted not to participate (main reason: too unwell (40%)). Compared to working hours, a participant was recruited every 14 h. Comparing characteristics, a trend towards better health and younger age was observed.

**Conclusion:**

ED-based recruitment offers the opportunity to include more diverse individuals in falls prevention. To achieve adequate sample sizes, flexibility in working days and hours of the research team are obligatory.

**Trial registration:**

DRKS00025949.

**Supplementary Information:**

The online version contains supplementary material available at 10.1186/s12877-023-04607-5.

## Background

In 2022, the global world guidelines for falls prevention (WFG) were updated. Besides a core set of recommendations, the authors call for multifactorial assessments in future care and research to not only determine an individual’s risk of future falls and fall-related injuries, but also tailor therapeutic efforts [1]. It may be particularly challenging to establish these assessments in the clinical setting of the emergency department (ED), which is characterized by crowding, hectic atmosphere, and high workload – yet often represents the initial contact for older adults with the healthcare system after a fall [2, 3]. Therefore, assessment protocols adapted to the situation in the ED are needed, which identify patients at highest risk for further falls and future functional loss. This aim is targeted by the SeFallED study currently ongoing in Oldenburg, Germany [4].

In general, older adults with a higher social status, healthier lifestyle, and less preexisting diseases are overrepresented in research studies [5]. An ED-based recruitment process, however, may alleviate this selection bias, as characteristics such as living in a rural area and low income are associated with frequent ED visits in older adults [6]. Therefore, it is a key focus of the study to recruit a sample representative of people commonly attending the ED. Different recruitment strategies for older adults and for falls prevention have been discussed before [7, 8], but appear to be particularly challenging to apply in the ED. Therefore, we present the success and challenges of an ED-based recruitment for an observational study on falls in older people presenting to the ED without consecutive admission to the hospital.

## Methods

The SeFallED study is a mixed-methods study with its main part being an observational prospective study of 24 months on older adults, who presented to the ED after a fall without subsequent hospitalization. Inclusion criteria were an age of ≥60 years, the ability to walk without another person’s support, and a life expectancy of more than three months [4]. The SeFallED study has been prospectively registered (Deutsches Register für klinische Studien, DRKS00025949), is in accordance with the Declaration of Helsinki, and was approved by the Medical Ethics Committee of the University of Oldenburg (number 2021–120).

### Recruitment process

At the beginning of the SeFallED study, a three-step recruitment process was developed following recommendations of previous research [7, 9]:

(1) A study nurse is present in the ED from Monday to Friday during regular working hours (8.30 am to 2.30 pm from Monday to Friday), screens all new admissions and addresses suitable individuals to obtain written consent for further contact via telephone. (2) Individuals are contacted via telephone within a week of their ED visit to schedule a first in-person-appointment within 4 weeks. (3) At the in-person-appointment at their home, individuals receive study documents and provide written informed consent for participation.

study documents and provide written informed consent for participation.

To maintain accuracy in addressing individuals in the ED, all those responsible for recruiting participants underwent comprehensive training. This training encompassed adherence to standard operating procedures (SOPs), collaboratively developed with the SeFallED study’s participatory research team, consisting of six older adults aged 66 to 84 years with prior experiences of falls [4].

### Recruitment centers

Participants were recruited in the Klinikum (KOL) and the Evangelisches Krankenhaus (EV) in Oldenburg, a town with 173.987 inhabitants in a rural region. The catchment area of the two hospitals includes the health care region “Weser-Ems” adding up to around 1.7 million people.

According to the structured quality reports of both hospitals from 2020, the KOL has more beds (around 830) in comparison to the EV (around 420), more employees and more medical departments [10, 11]. In 2020, the EV treated around 56.000, the KOL 126.500 patients. The two hospitals are responsible for trauma surgery care in Oldenburg [12]. The EV is directly located in the city center of Oldenburg with the KOL being around 5 km away from the city center in a district called Kreyenbrück. Whereas the proportion of older adults above 65 years of age is rather similar in both districts (around 20%), more individuals that are dependent on social welfare and more people without German citizenship (Kreyenbrück: 21.6%; city center: 7.2%) as well as third-generation migrants (Kreyenbrück: 43.8%; city center = 17.5%) live in Kreyenbrück.

### Outcomes and statistical analyses

The outcomes are presented in line with the three-step recruitment process. At first, the recruitment rate in the KOL will be analyzed. Total admissions to the ED outside of working hours of the study team between 15/11/2021–30/07/2023 are presented sorted by weekday and time of the day. Furthermore, characteristics of patients (age, sex, presentation to the ED (self-presentation, presentation via ambulance)) admitted to the ED outside of working hours will be compared with patients approached by the study team to address representativity of the sample. These will be divided into three groups: (1) patients addressed but immediately declining participation, (2) patients declining participation after being contacted via telephone, (3) participants enrolled in the study. Between-group comparisons were performed using chi-square test for categorical variables and Kruskal-Wallis test for continuous variables.

In a second step, between-group comparisons were carried out for participants addressed by the study team. Besides the aforementioned characteristics, education (low, middle, high), place of birth (PoB; Germany/Other) and level of care (Yes/No) were assessed for these three groups via interview. Level of care (known as ‘Pflegegrad’ in German) is a classification that evaluates an individual’s need for support due to disabilities or the requirement for care. This classification is determined by assessing various criteria, including physical or mental health conditions, independence in daily activities, cognitive abilities, and the need for assistance. Analysis of variance (ANOVA), in case of non-normally distributed data, a Kruskal-Wallis test, was used to analyze differences between the three groups. In case of significant main effects, post hoc pairwise comparisons were conducted using Bonferroni correction for multiple pairwise comparisons. Due to data security regulations, data of patients outside of working hours and patients immediately declining to participate, were only accessible for the KOL and not the EV.

Thirdly, characteristics of patients, who declined to participate after telephone contact, and participants enrolled in the study were compared across and between the two EDs. A two-way ANOVA was performed to analyze the effect of group (declined to participate after telephone contact versus patients enrolled) and emergency department (KOL versus EV) on age. Categorical variables (sex, presentation to the ED, education, PoB, level of care, and living situation (independent/care home)) were compared using chi-square test. Care home refers to to a facility providing nursing and long-term care services for individuals requiring assistance with daily living due to age, illness, or disability. Furthermore, the number of calls to participants until either dropout or enrolment were calculated and analyzed using point-biserial correlation coefficient to determine the association between repetitive calls and enrollment. Lastly, the study team asked patients for their main reason for dropping out, which was categorized into “too unwell”, “no interest”, “no time”, “caregiver/relative declines”, “hospital admission/ death” and “others”. Participants, who could not be reached by the study team via telephone and did not react to letters sent to them, were categorized as “no contact”. Furthermore, we provide descriptive data for the individuals successfully recruited into the study. This data includes the count of preexisting health conditions, their functional ability classified as Robust, postRobust, preFrail, and Frail according to the Longitudinal Urban Cohort Ageing Study Functional Ability Index (LUCAS-FI) [13], as well as their score from the Short Physical Performance Battery Test [14], which ranges between 0 and 12 points. SPSS 29 was used for all analyses with α set at 0.05. Continuous variables were expressed as mean ± SD.

## Results

### Changes in recruitment strategy

Recruitment started at the KOL on 15/11/2021, however, a second hospital (EV) was added from 17/1/2022 due to dissatisfying recruitment numbers. Further, an inclusion criterion was adjusted to allow individuals, who lived up to 40 km away from the study center to be included in the study, compared to the originally defined 20 km. With these changes, average recruitment per month was increased from 7.5 to 15.7 participants. Working hours of the study team were increased by additionally employing research assistants. Thus, study personnel was present for a minimum of 24 h and a maximum of 68 h a week between 17/01/2022 and 31/08/2023.

### Eligible and recruited participants in the KOL

In total, 1518 patients presenting to the ED of the KOL between 15/11/2021–31/07/2023 fulfilled the inclusion criteria of the SeFallED study. The study team addressed 377 (24.8%) of these individuals with 106 patients immediately declining after being contacted in the ED (7.0% of all admissions; 28.0% of individuals addressed by the study team). Of the patients signing up for further contact (n = 271, 17.9% of all admissions), 141 (9.3% of all admissions; 37% of all individuals addressed by the study team) were recruited.

On weekdays, most participants were missed on Wednesdays between 2.30pm–10pm (n = 127), where most general practitioners are closed in Germany. In general, most patients were missed between 6pm–10pm (n = 455). Supplementary Table 1 provides a detailed overview of patients missed by day and time.

Presentation to ED differed between the groups with bonferroni corrected post hoc tests revealing that individuals, who declined to participate during telephone contact were more strongly represented in the group admitted by ambulance (*p* < 0.001). No further differences were detected (Table 1).

**Table 1:** Patient characteristics stratified by group (total admissions, decline to participate in the ED, decline to participate telephone contact, participants)

		total admissions	decline to participate in the ED	decline to participate after telephone contact	participants	Statistics
**Number of participants**		1141	106	130	141	/
**Age, years** (mean ± SD)		78.2 ± 10.5	78.6 ± 11.1	79.7 ± 9.3	76.7 ± 9.0	0.076
**Sex, n** (males/females)		429/712	35/71	49/81	50/91	0.787
**Presentation to ED, n**	Self-presentation	486	44	40	73	**0.011**
	Ambulance	627	61	86	68	
	Missing	28	1	4	0	

ED = emergency department

Table 2 shows the characteristics of the participants addressed in person in the ED of the KOL. Significant main effects were revealed between the groups for age, living status, presentation to the ED, education, and level of care, but not for PoB and sex. Post hoc test showed that participants enrolled in the study were younger, less likely to be taken to the ED by ambulance and more likely to have a level of care in comparison to individuals, who declined after being contacted by telephone, but not to those declining to participate in the ED. Participants immediately declining to participate were less likely to have a high education in comparison to the other two groups according to Bonferroni corrected post hoc test. Furthermore, post hoc tests revealed that participants enrolled in the study lived less often in a care home compared to the other groups.

**Table 2:** Patient characteristics stratified by group (decline to participate in the ED, decline to participate telephone contact, participants)

		Decline to participate in the ED (1)	Decline to participate after telephone contact (2)	Participants (3)	Statistics	Post hoc		
						1–2	1–3	2–3
**Number of participants**		106	130	141	/			
**Age, years (mean** ± SD)		78.6 ± 11.1	79.7 ± 9.3	76.7 ± 9.0	**0.019**	0.494	0.183	**0.020**
**Sex, n** (males/females)		35/71	49/81	50/91	0.757	/	/	/
**Place of birth, n** (Germany/Other (missing))		38/7 (61)	56/10 (64)	81/6 (54)	0.185	/	/	/
**Level of care, n** (No/Yes (missing))		56/31 (19)	54/49 (27)	91/35 (15)	**0.008**	0.097	0.223	**0.002**
**Living status, n** (independent/ care home (missing))		64/15 (27)	89/20 (21)	120/4 (17)	**< 0.001**	0.993	**< 0.001**	**< 0.001**
**Presentation to ED, n**	Self-presentation	44	40	73	**0.005**	0.131	0.157	**0.001**
	Ambulance	61	86	68				
	Missing	1	4	0				
**Education, n**	Low	34	65	62	**0.001**	**0.010**	**0.005**	0.132
	Middle	24	19	28				
	High	2	11	25				
	Missing	46	35	26				

ED = emergency department

### Recruitment in the KOL and EV

When comparing working hours and recruitment success, the study team had to be present in the EDs for an average of 14 h per participant. Recruitment rate was significantly different between the two EDs with 62% of individuals, who provided initial consent for contact, being enrolled in the study in the EV and only 52% in the KOL. Comparing the populations of the EDs with each other, two-way ANOVA revealed that patients in the EV were younger. Furthermore, individuals declining to participate after telephone contact were significantly older across EDs with a significant within difference solely for individuals of the KOL but not EV according to Bonferroni corrected post hoc tests. Presentation to the ED was significantly different across the EDs, with a higher percentage (57%) of individuals being transported by ambulance to the ED of the KOL than to the EV (40%).

Whereas no significant difference in sex, PoB, or education was observed, level of care differed within each ED with less individuals with a level of care being enrolled in the study in the KOL and the EV. Additionally, individuals, who declined to participate after being contacted via telephone, were more likely to live in a care home in the KOL, but not in the EV (Table 3). Among the recruited individuals, 33% were categorized as Frail, 42% as Robust, 17% as preFrail and 7% as PostRobust based on the LUCAS-FI. These individuals showed a mean SPPB score of 8.9, ranging from 1 to 12 points. Additionally, they had an average of 2.2 preexisting health conditions, ranging from 0 to 6.

**Table 3:** Patient characteristics stratified by emergency department (KOL and EV) and group (decline to participate telephone contact, participants)

		KOL				EV				Statistics (between EDs)
**Individuals addressed, n** (declined/recruited)		271 (130/141)				230 (86/144)				**0.017**
		*Total*	*Declined*	*Recruited*	*Statistics*	*Total*	*Declined*	*Recruited*	*Statistics*	
**Age, years** (mean ± SD)		78.1 ± 9.2	79.7 ± 9.3	76.7 ± 8.9	**0.015**	75.8 ± 9.4	76.5 ± 9.9	75.3 ± 9.0	0.339	**0.008**
**Sex, n** (male/female)		99/172	49/81	50/91	0.703	73/157	27/59	46/98	0.931	0.151
**Place of birth, n** (Germany/Other (missing))		137/16 (118)	56/10 (64)	81/6 (54)	0.098	116/14 (100)	42/4 (40)	74/10 (60)	0.572	0.542
**Level of care, n** (No/Yes (missing))		145/84 (42)	54/49 (27)	91/35 (15)	**0.002**	106/49 (75)	31/25 (30)	75/24 (45)	**0.009**	0.306
**Living status, n** (independent/ care home (missing))		209/24 (38)	89/20 (21)	120/4 (17)	**0.001**	144/13 (73)	53/8 (25)	91/5 (48)	0.080	0.504
**Presentation to ED, n** (self-presentation/ ambulance (missing))		113/154 (4)	40/86 (4)	73/68 (0)	**0.001**	134/92 (4)	43/40 (3)	91/52 (1)	0.093	**0.001**
**Education**	Low	127	65	62	0.068	58	21	37	0.499	0.260
	Middle	47	19	28		33	8	25		
	High	36	11	25		16	5	11		
	Missing	61	35	26		123	52	71		

ED = emergency department; KOL = Klinikum Oldenburg; EV = Evangelische Krankenhaus Oldenburg

### Phone contact and reasons to decline

The study team required an average of 2 telephone calls to reach the individuals, who consented to being contacted. If a person was unable to make a decision regarding participation in the study, they were offered the option to be contacted again at a later date, which led up to a maximum of 12 tries of reaching participants. In case of numerous contacts, biserial point correlation revealed that declining to participate was positively associated with the number of contacts (r = 0.552, *p* < 0.001).

**Fig. 1:**
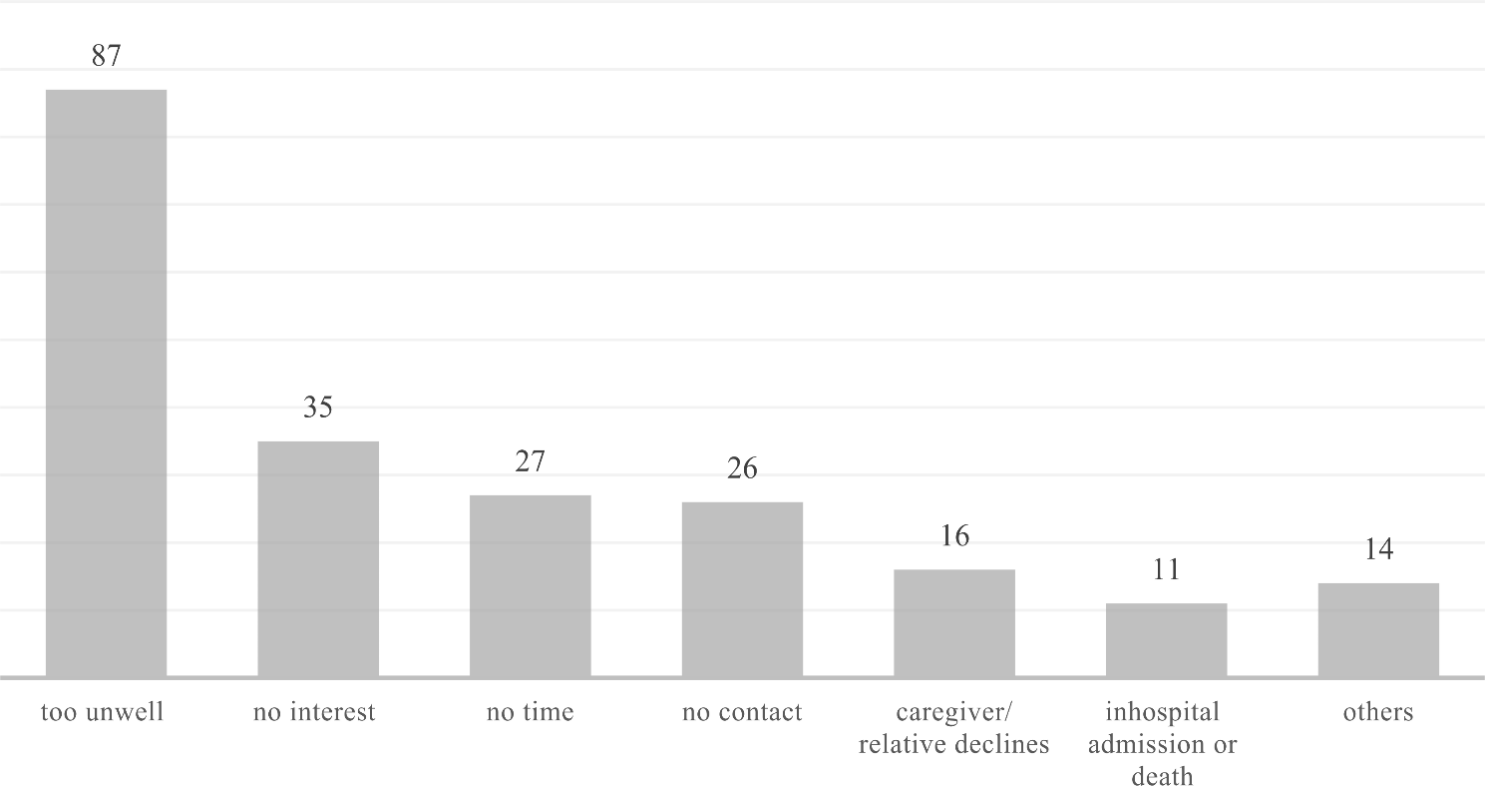
Reasons for declining to participate during telephone contact

The most common reason to decline participation during telephone contact was being “too unwell” (Fig. 1). This was followed by “no interest”, lack of time and being unable to reach the participants within four weeks after their initial ED visit. In less than 10% of the cases, caregivers or relatives decided against participation, followed by “hospital admission/death”. 7% of all participants gave other reasons for dropping out (e.g., fear of Corona virus, moving house).

## Discussion

ED-based recruitment of participants has been challenging in this study. Between 15/11/2021–31/07/2023 a total of 288 participants were enrolled by recruiting in two different EDs. A trend towards a lower age, higher education and better health status was observed in the recruited sample, nevertheless, recruitment in a clinical setting such as the ED may still present an opportunity to approach a heterogenous group of people more representative to real life.

The recruitment rate between individuals approached in person and recruited, which was only possible to assess in one of the EDs due to data security regulations, was 37%. Compared to previous interventional studies in falls prevention (38.9–84.5%) [8] and general research (median of 41.4%) with older adults, the participation rate is only slightly lower [7]. As 16% of the individuals addressed in the SeFallED study dropped out due to lack of interest, it may be hypothesized that the observational design accounts for some difficulties in recruitment. Previous studies have assessed that the lack of a personal benefit may act as a barrier to participation in research [15, 16], which was also quoted by the SeFallED study’s participatory research team [4]. Even though the participatory research team advised study personnel to hand out individual results to participants as feedback throughout the course of the study, a somewhat negative “burden:benefit” ratio was inevitable due to the observational character of the study [15]. Therefore, it may be speculated that the “burden:benefit” ratio would be positively changed by offering structured falls prevention tailored to individual needs, barriers and preferences in addition to observing functional trajectories. However, future research is warranted to confirm such speculations. The ongoing Covid-19 pandemic may have further affected willingness to participate in research studies, as experts advised older adults to minimize contacts, especially during winter months [17–19].

Recruiting a diverse sample of older adults in regard to sex, age, PoB, health, and socioeconomical status in health-related research is challenging according to previous research [20–22], but may be alleviated by an ED-based recruitment [6]. Moreover, previous research has indicated the significance of race/ethnicity concordance between the research team and the studied population in fostering inclusivity [23, 24]. In this study, aligning the demographics of the individuals engaged in recruitment with the target population in terms of sex and PoB potentially facilitated the inclusion of individuals born outside of Germany. However, future research ought to investigate the impact of research team demographics on recruitment efficacy.

In the ongoing study, it was particularly difficult to recruit individuals living in care homes and those admitted to the ED via ambulance. Furthermore, individuals with a higher education were more likely to participate in the study, which is in line with previous health-related research [25]. Nevertheless, most participants enrolled in the study had been in school for less than 10 years, which indicates a low school education and emphasizes the added value of an ED-based recruitment. To further engage individuals living in care homes in research, it may be useful to establish collaborations between care providers and the research team, which has been discussed as a facilitator [26]. However, this approach would increase the complexity of the recruitment process and require additional resources (e.g., more study personnel). The recruited sample is diverse in terms of the comprehensive points scored in the SPPB, the count of diagnoses, and their categorization according to the LUCAS-FI. Thus, recruiting from EDs offers an opportunity not only to include a heterogenous group of individuals but also to specifically target frail and multimorbid individuals.

Previous research indicated, that self-referred patients are less severely ill than individuals arriving by ambulance in EDs [27, 28]. Gries and colleagues retrospectively analyzed data of 34,178 patients, who presented to the ED in Leipzig, Germany, and showed that self-referred patients had the lowest likelihood of being admitted to hospital [29]. Therefore, it may be speculated, that individuals, who experienced a more severe fall and were consequently taken to hospital by ambulance, may need more time to recover than individuals presenting themselves to the ED. As being too unwell was the most quoted reason to decline participation during telephone contact, it might be speculated that recruitment would benefit from extending the 4 weeks window to conduct a geriatric assessment to also recruit individuals more affected by their fall. However, the first home assessment evaluates the acute effects of a fall. and, thus, needs to be conducted as soon as possible. To confirm our speculations, future research may look into associations between severity of the fall and recruitment success.

As the WFG recommends timely multifactorial assessments to establish tailored follow-ups for individuals presenting to the ED [1], a solution may be to integrate multifactorial assessments into clinical practice in the ED. However, a previous qualitative research study, analyzing physical therapy consultations for falls in the ED, concluded that resources for such interventions are scarce [30]. Due to an increasing shortage of staff, particularly nurses, in clinical settings [31], further worsening due to the Covid-19 pandemic [32], it is more than questionable if resources allow to integrate extensive, multifactorial assessments in clinical practice. A solution may be to officially prescribe a follow-up appointment with the general practitioner or specific fall centers, which assess risk factors and organize secondary prevention approaches [33]. Such fall centers may also act as a starting point for recruiting individuals at high-risk, as it would allow to approach persons outside of the ED’s busy environment and in a non-emergency condition [34, 35]. Nevertheless, financial and personnel resources are needed to establish such a structure and prescriptions need to target all individuals. Future research needs to evaluate the efficacy of such an approach.

Challenges in staffing may also explain difficulties experienced within this study, as different strategies such as involving the medical staff in the recruitment process [36] could not be established successfully. Even though the research team organized small celebrations for milestones (e.g., handing out muffins; Christmas cards) to actively involve staff, presented and explained the study in staff meetings and put up posters, recruitment through staff outside the research team’s working hours was not possible. Placing leaflets, which were developed together with the participatory research team [37], in the waiting as well as examination rooms led to only three individuals actively addressing the research team. Therefore, it is key to directly talk to potential participants, especially in such a physically and psychosocially demanding situation [38], which warrants enough study personnel in future research to cover more times (e.g., Wednesdays and Saturdays and times between 6pm-10pm).

Recruitment numbers revealed differences between EDs, which may partly be explained by differences in health status (i.e., presentation to the ED) and age, but also by structural differences of the city districts as well as the size of the hospitals. According to official reports by the city of Oldenburg, more individuals dependent on social welfare and more people without German citizenship as well as third-generation migrants live close to the KOL [39]. Therefore, it may be speculated that in line with prior research a lower socio-demographic might explain differences between hospitals [20–22]. Moreover, crowding in EDs is associated with reduced patient satisfaction [40], which may occur more frequently in the bigger hospital (KOL) and interfere with participation in a research project. Further analyses are needed, which take quantitative and qualitative data into account, to determine differences across EDs. In regard to recruiting a diverse sample of participants the use of more than one ED seems advisable.

### Strengths and limitations

We were not able to collect full data sets due to incomplete reporting in electronic hospital forms and by patients, who had a relatively low level of trust to share personal information when approached in the ED [41]. Due to data security regulations and ethical concerns such as the traumatic situation in the ED it was not possible to assess the specific type of injury that the individuals presented with at the ED. As severe fractures and head traumas would have required hospitalization, it can be speculated that somewhat similar injuries such as lacerations, mild concussions, or contusions were present in the patients addressed by the study team. Nevertheless, future studies may focus on classifying the types of injuries among individuals presenting to the ED following a fall. Furthermore, total admissions could only be assessed in one of the hospitals due to different data protection regulations. Nevertheless, the data obtained, provides important information about patient availability in the ED addressing research priorities of the WFG such as assessing high-risk groups in challenging settings. This is needed to better understand this population and develop data driven fall prediction models and prevention programs. Regular working hours of our study nurses were from 8:30 am to 2:30 pm, which resulted in missing potential participants, notably in the evenings as indicated by our analyses. Despite employing research assistants to cover additional shifts in the ED, optimizing future recruitment may involve a shift to later working hours. However, given the prevalent female dominance among nursing staff, and considering that female employees often manage multiple responsibilities both at work and home, adjusting to later shifts could pose significant challenges [42–44]. This shift might not only hinder the recruitment of qualified personnel but also exacerbate gender disparities. Employing (medical) students as research assistants emerges as a potentially favorable solution to enhance recruitment in future studies while ensuring equal opportunities.

## Conclusion

Recruitment in the ED provides the opportunity to include more diverse individuals in falls prevention. To reach adequate sample sizes, flexibility in working days and working hours of the research team are obligatory. This requires bigger research teams (benefit of face-to-face contact/ low support of regular medical staff), for whom funding needs to be provided. Furthermore, studies may benefit from interventional study designs to increase recruitment rates.

### Electronic supplementary material

Below is the link to the electronic supplementary material.

Supplementary Material 1

## Data Availability

The dataset used and analyzed during the current study are available from the corresponding author upon reasonable request.

## Abbreviations

ANOVA: Analysis of Variance
ED: Emergency Department
EV: Evangelisches Krankenhaus
KOL: Klinikum Oldenburg
SD: Standard Deviation
SeFallED: Sentinel Fall Presenting to the Emergency Department
WFG: World Guidelines for Falls Prevention
