# Supplementary material for: Recruiting patients for falls prevention in the emergency department – worth the challenge
Source: BMC Geriatr. 2023 Dec 21;23:880. doi: 10.1186/s12877-023-04607-5 (PMC10740331; doi:10.1186/s12877-023-04607-5)
Supplement: Supplementary file 1 — Supplementary Material 1 [file 12877_2023_4607_MOESM1_ESM.docx]

Supplementary Table 1: Numbers of patients missed in the KOL sorted by day and time of the day

| **Day** | **Total** | **7.30am – 2.30pm** | **2.30pm – 6pm** | **6pm – 10pm** | **10pm – 7.30am** |
| --- | --- | --- | --- | --- | --- |
| **Monday** | 133 | 28 | 19 | 66 | 20 |
| **Tuesday** | 145 | 19 | 37 | 68 | 21 |
| **Wednesday** | 191 | 35 | 50 | 77 | 29 |
| **Thursday** | 166 | 25 | 29 | 81 | 30 |
| **Friday** | 137 | 21 | 37 | 48 | 31 |
| **Saturday** | 187 | 47 | 54 | 64 | 22 |
| **Sunday** | 182 | 54 | 50 | 50 | 28 |
| **Total** | 1141 | 229 | 276 | 455 | 181 |

KOL = Klinikum Oldenburg
